# Sustained Release Varnish of Chlorhexidine for Prevention of Biofilm Formation on Non-Absorbable Nasal and Ear Sponges

**DOI:** 10.3390/pharmaceutics18010096

**Published:** 2026-01-12

**Authors:** Sari Risheq, Athira Venugopal, Andres Sancho, Michael Friedman, Irit Gati, Ron Eliashar, Doron Steinberg, Menachem Gross

**Affiliations:** 1 Department of Otolaryngology-Head and Neck Surgery, Hadassah Medical Center, Jerusalem 9112102, Israel; 2 The Biofilm Research Laboratory, Faculty of Dental Medicine, Hebrew University, Jerusalem 9190500, Israeldorons@ekmd.huji.ac.il (D.S.); 3 Institute for Drug Research, School of Pharmacy, Hebrew University, Jerusalem 9190500, Israel; 4 Faculty of Medicine, Hebrew University, Jerusalem 9190500, Israel

**Keywords:** chlorhexidine, sustained-release varnish, Merocel sponge, biofilm prevention, *Staphylococcus aureus*, *Pseudomonas aeruginosa*

## Abstract

**Background**: Non-absorbable polyvinyl alcohol sponges (Merocel) are widely used in otolaryngology for nasal and ear packing but are prone to bacterial colonization and biofilm formation, which may increase infection risk and drive frequent use of systemic antibiotics. Sustained-release drug delivery systems enable prolonged local antiseptic activity at the site of packing while minimizing systemic exposure. **Methods**: We developed a sustained-release varnish containing chlorhexidine (SRV-CHX) and coated sterile Merocel sponges. Antibacterial, in vitro, activity against Staphylococcus aureus and Pseudomonas aeruginosa was evaluated using kinetic diffusion assays on agar, optical density (OD_600_) measurements of planktonic cultures, drop plate, ATP-based viability assays, biofilm analysis by MTT metabolic assay, crystal violet bio-mass staining, high-resolution scanning electron microscopy (HR-SEM), and spinning disk confocal microscopy. **Results**: SRV-CHX-coated sponges produced sustained zones of inhibition on agar plates for up to 37 days against *S. aureus* and 39 days against *P. aeruginosa*, far exceeding the usual 3–5 days of clinical sponge use. Planktonic growth was significantly reduced compared with SRV-placebo, and a bactericidal effect persisted for up to 16 days for S. aureus and 5 days for *P. aeruginosa* before becoming predominantly bacteriostatic. Biofilm formation was markedly inhibited, with suppression of metabolic activity and biomass for at least 33 days for *S. aureus* and up to 16 days for *P. aeruginosa*. HR-SEM and confocal imaging confirmed sparse, discontinuous biofilms and predominance of non-viable bacteria on SRV-CHX-coated sponges compared with dense, viable biofilms on the placebo controls. **Conclusions**: Coating Merocel sponges with SRV-CHX provides prolonged antibacterial and anti-biofilm activity against clinically relevant pathogens. This strategy may reduce dependence on systemic antibiotics and improve infection control in nasal and ear packing applications in otolaryngology.

## 1. Introduction

Non-absorbable sponges such as Merocel play an important role in otolaryngology, with wide applications in both the nasal cavity and the external ear canal. In the ear canal, Merocel is commonly used for the management of acute otitis externa, chronic otitis externa, necrotizing otitis externa, and post-operative packing [1,2]. In the nasal cavity, it is frequently applied for epistaxis control [3], post-surgical packing, and in specific cases such as septal hematoma or abscess drainage [4,5].

Otitis externa, also known as “swimmer’s ear,” is one of the most frequent conditions in ENT practice. Its severity ranges from mild localized infection to necrotizing otitis externa, which can cause significant pain and morbidity [6]. The disease affects both children and adults, substantially impacting quality of life [7]. In the United States, acute otitis externa (AOE) accounts for ~2.4 million healthcare visits annually (8.1 visits per 1000 population) and an estimated cost of $500 million [8]. Management typically involves topical antibiotics, with or without corticosteroids [1], although systemic therapy may be required in severe cases [8]. The majority of cases are bacterial, most frequently due to *Pseudomonas aeruginosa* and *Staphylococcus aureus*, though polymicrobial infections are also common [6,9,10].

Epistaxis is another common ENT emergency condition [3]. Etiologies include trauma, hypertension, vascular abnormalities, infections, inflammatory conditions, neoplastic disease, and certain medications. It occurs more frequently during colder months [11]. Anatomically, epistaxis is classified as anterior—most often from Kiesselbach’s plexus (Little’s area)—or posterior, which is less common but more severe [12]. Management ranges from compression to nasal packing and, in refractory cases, arterial ligation or embolization [13]. However, nasal packing creates an environment favorable for bacterial colonization and biofilm formation. Consequently, systemic antibiotics are often prescribed, although this practice remains controversial [8,14,15]. Merocel is also used post-operatively to reduce bleeding and in special cases such as septal hematoma or abscess drainage.

Thus, non-absorbable sponges such as Merocel are consistently challenged by bacterial colonization, biofilm formation, and subsequent reliance on systemic or topical antibiotics. To address this issue, we hypothesized that biofilm accumulation could be inhibited by coating Merocel with a sustained-release varnish (SRV) containing an antiseptic agent, thereby prolonging local antimicrobial activity and reducing systemic antibiotic exposure.

**Figure 1:**
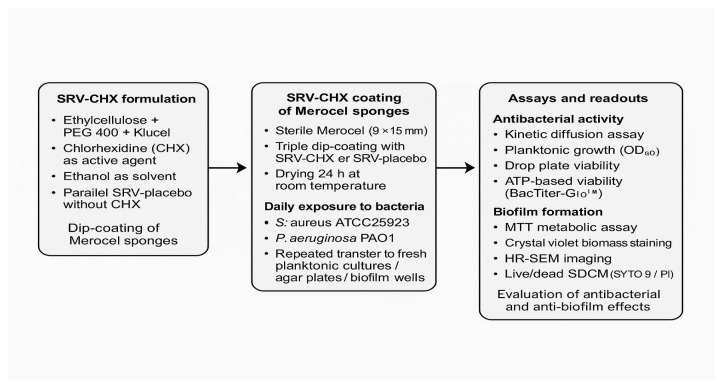
Overview of experimental design. A schematic representation of the SRV-CHX formulation process, sponge coating procedure, bacterial exposure regimen, and all microbiological and imaging readouts used in this study.

Chlorhexidine (CHX), a quaternary ammonium antiseptic, has been successfully applied to multiple medical devices [16]. It is particularly suited for incorporation into SRV formulations and was therefore selected for this study. Recent investigations of SRV-CHX in otolaryngology have shown promising results, including effective prevention of biofilm formation on sino-nasal stents [17] and voice prostheses [18]. An overview of the experimental workflow is schematically illustrated in Figure 1.

## 2. Materials and Methods

### 2.1. Sustained Release Varnish (SRV) Preparation

All chemicals and materials used in this study were of analytical or pharmaceutical grade. Chlorhexidine (CHX), ethylcellulose (Ethocel N-100), polyethylene glycol 400 (PEG-400), Klucel (hydroxypropyl cellulose), and ethanol were obtained from Sigma-Aldrich (St. Louis, MO, USA) or Hercules Inc (Wilmington, DE, USA). The sustained-release varnish (SRV-CHX) was formulated using a modified protocol adapted from Friedman et al. [19], optimized to ensure strong adhesion to the spongiform Merocel surface. Ethylcellulose and PEG-400 formed the polymeric matrix, CHX served as the active antimicrobial component, and ethanol was used as the solvent. Klucel was added to improve film formation and mechanical stability. A placebo varnish (SRV-Placebo) was prepared using the same formulation but without the inclusion of CHX.

### 2.2. Coating of the Sponge

The coating was conducted in the dip–dry method. A sterile sponge (Merocel, Medtronic, Minneapolis, MN, USA; 9 mm × 15 mm) was dipped in the SRV-CHX and SRV-Placebo solution and allowed to dry at room temperature for 24 h. Dipping was repeated three times for each sponge. The amount of SRV-CHX was calculated by weighing the sponge before and after coating with the SRV-CHX. In this study, the effect of SRV-CHX was compared to SRV-Placebo.

### 2.3. Bacterial Strains

The bacterial strains that were used included *S. aureus* ATCC 25923 and *P. aeruginosa* PAO1 (from the stocks of Prof. Doron Steinberg’s Lab), as a model for strongly adhering bacteria to the sponge. Bacteria were cultivated overnight at 37 °C in tryptic soy broth (TSB) (Acumedia, Neogen, Lansing, MI, USA). Fresh bacterial cultures were prepared daily. The OD_600nm_ of the bacteria used for each study was identical (OD_600nm_ of 0.02).

### 2.4. Bacterial Exposure Assay

The SRV-CHX and SRV-Placebo-coated sponges were immersed in 3 mL of tryptic soy broth (TSB; HiMedia Laboratories Pvt., Ltd., Maharashtra, India) supplemented with 1% glucose (TSBG) and 30 µL of an overnight bacterial culture. The samples were incubated for 24 h at 37 °C. Following incubation, the sponges were transferred daily to fresh TSBG containing a newly prepared bacterial culture to ensure continuous exposure.

### 2.5. Non-Absorbable Sponge (Polyvinyl Alcohol [MEROCEL])

**Figure 2:**
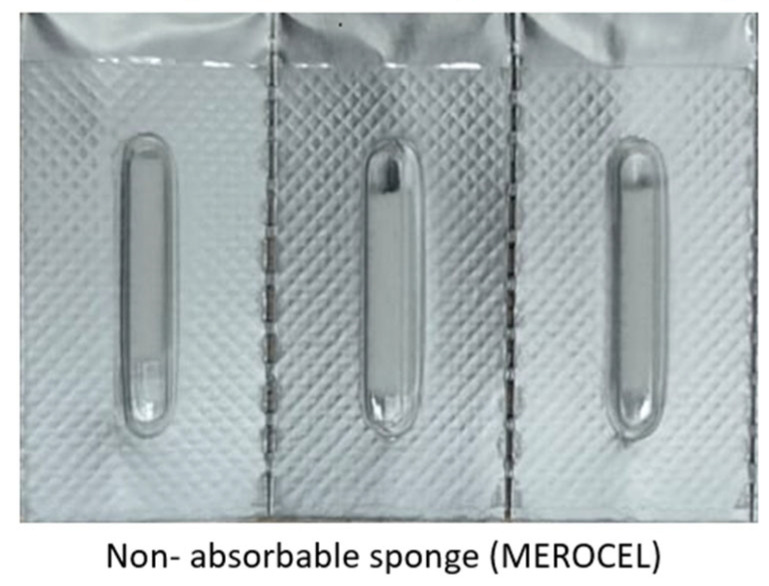
Non-absorbable polyvinyl alcohol sponge (Merocel) used in this study.

Several sizes of non-absorbable sponges (Merocel) were used in the market. For this study, we decided to use the sterile sponge (9 mm × 15 mm) made by Medtronic (Jacksonville, FL, USA) throughout the study, due to the sterility and the equal size, shape, and weight of the sponges, which provided comparable data (Figure 2). 

### 2.6. Determination of Anti-Bacterial Activity

#### 2.6.1. Kinetic Diffusion Sensitivity Test

Sponges (Merocel) coated with SRV-CHX and SRV-Placebo were repeatedly placed on tryptic soy agar plates pre-seeded with *S. aureus* or *P. aeruginosa* and incubated at 37 °C for 24 h [20]. The zone of inhibition around the placed sponges (Merocel) was measured daily, and the sponges (Merocel) were transferred aseptically to new bacteria-seeded agar plates for further incubation. This procedure was conducted for up to 40 days. The zone of inhibition was quantified as the clear area (cm^2^) around the sponge (r_1_ × r_2_ × π) after subtracting the area of the sponge. r_1_ and r_2_ are the radius of the vertical and horizontal direction, respectively.

#### 2.6.2. Measuring the Effect of Packing (Merocel) on Planktonic Bacterial Growth

The coated sponges (Merocel) were repeatedly incubated in 3 mL of TSBG medium with 30 µL of an overnight bacterial culture in 15 mL tubes for 24 h. The optical density (OD) at 600 nm was measured using a spectrophotometer, and the amount of bacteria in the supernatant was determined relative to TSBG incubated with the sponge in the absence of bacteria. The following formula was used for the calculations: % Viability = (OD_SRV-CHX_ − OD_background_)/(OD_SRV-Placebo_ − OD_background_) × 100%.

#### 2.6.3. Drop Plate Method to Determine Bacterial Viability

The agar plate was divided into sectors, with each sector receiving two 10 µL drops of the bacterial suspension that had been exposed to either SRV-CHX or SRV-Placebo-coated sponges (Section 2.6.2). The plates were subsequently incubated upside down at 37 °C overnight to visualize any bacterial growth.

#### 2.6.4. BacTiter-Glo^TM^ Microbial Cell Viability Assay

The BacTiter-GloTM microbial cell viability assay (Promega Corporation, Madison, WI, USA) was used to determine the relative number of viable bacterial cells in culture based on the quantitation of intracellular ATP. One hundred µL of each sample was mixed with 100 µL of the reagent in 96-flat bottom plates (Greiner Bio-One, μClear white clear bottom), and after mixing for 5 min on an orbital shaker, the luminescence was recorded using the M200 Tecan plate reader (Tecan Trading AG, Männedorf, Switzerland). The following formula was used for the calculations: % Viability = (RLU_SRV-CHX_ − RLU_background_)/(RLU_SRV-Placebo_ − RLU_background_) × 100%, where RLU is the relative luminescence units.

### 2.7. Determination of Biofilm Biomass

To study the effect of SRV-CHX-coated sponges on biofilm formation, the sponges were incubated in 12-well tissue culture plates in 3 mL of TSBG, to which 30 μL of an overnight bacterial culture was added. The biofilms formed at the surface of the wells were rinsed gently with PBS, followed by MTT metabolic assay (see Section 2.7.1 for details) or crystal violet (CV) staining for total biofilm mass (see Section 2.7.2 for details). The sponges were daily transferred to new tissue culture plates and exposed to a new, fresh bacterial culture. This was repeated until the anti-biofilm effect ceased.

#### 2.7.1. Biofilm Metabolic Assay

The metabolic activity of biofilms formed was determined using the MTT method [21]. The biofilms were rinsed gently with PBS and then exposed to 2 mL of 0.5 mg/mL MTT (Sigma, St. Louis, MO, USA) for 1 h, and the amount of tetrazolium formed was measured in an M200 Infinite plate reader (Tecan Trading AG, Männedorf, Switzerland) at an optical density of 570 nm after being dissolved in 1 mL dimethyl sulfoxide (DMSO).

#### 2.7.2. Crystal Violet (CV) Staining of Biofilms

The biofilms were stained for 20 min with 0.25% crystal violet solution that was prepared from a 1% Gram’s crystal violet stain solution (Merck, EMD Millipore Corporation, Billerica, MA, USA) diluted in DDW [22]. The stained biofilms were rinsed gently several times in DDW, and the intensity of the CV staining was measured in an M200 Infinite plate reader (Tecan Trading AG, Männedorf, Switzerland) at an optical density of 595 nm after being dissolved in 2 mL 33% acetic acid.

#### 2.7.3. High-Resolution Scanning Electron Microscope (HR-SEM) Imaging of Biofilms

The biofilm formed on the sponges (Merocel) was rinsed gently twice with PBS and twice with DDW before fixation with 4% glutaraldehyde in DDW for 20 min. After fixation, the samples were washed with DDW and dried before coating with iridium and visualized using a MagellanTM 400 L High-Resolution Scanning Electron Microscope (FEI Company, Hillsboro, OR, USA) at 200×–20,000× magnification [23]. 

#### 2.7.4. Spinning Disk Confocal Microscope (SDCM) Imaging of Biofilms

The sponges were daily exposed to freshly prepared planktonic growing bacteria for 7 days. During the last day of incubation, the sponges were incubated in 8-well ibidi glass slides, and the biofilms formed on the slide surfaces were rinsed gently twice with PBS and the attached biofilms were stained with the live/dead SYTO 9/propidium iodide (PI) BacLight fluorescent dyes (Life Technologies, Carlsbad, CA, USA) for 20 min in the dark [24]. After washing with PBS, the samples were visualized using a spinning disk confocal microscope (Nikon Yokogawa W1 Spinning Disk with 50 µm pinholes, Nikon Corporation, Tokyo, Japan). The fluorescence emission was observed using a 10×/0.45 lens (Plan-Apochromat Lambda) and captured by an SCMOS ZYLA camera. SYTO 9 fluorescence was measured using 488 nm excitation and 515 nm emission filters (Chroma Technology Corp., Bellows Falls, VT, USA), while PI fluorescence was measured using 543 nm excitation and 570 nm emission filters (Chroma Technology Corp., Bellows Falls, VT, USA). The biofilm depth was examined by generating optical sections that were acquired at spacing steps of 2.5 µm. The relative amount of *S. aureus* and *P. aeruginosa* viable cells in each sample was computerized as a color-appropriated fluorescence intensity using the NIS-Element AR software. The data were displayed as the amount of *S. aureus* or *P. aeruginosa* viable cells in each layer of the biofilm (2.5 µm). The percentage of the total biomass of viable cells in biofilm formed in the presence of SRV-CHX/SRV-Placebo was calculated as the integral of the curve and compared to the SRV-Placebo control. Experimental and placebo groups were compared in parallel under the same experimental setup.

### 2.8. Statistical Analysis

The statistical mean of three or four independent experiments was calculated. Statistical analysis was performed using the Student’s *t*-test, with a *p* value < 0.05 considered statistically significant.

## 3. Results

### 3.1. SRV-CHX-Coated Sponges Caused Bacterial Clearance for a Prolonged Time

**Figure 3:**
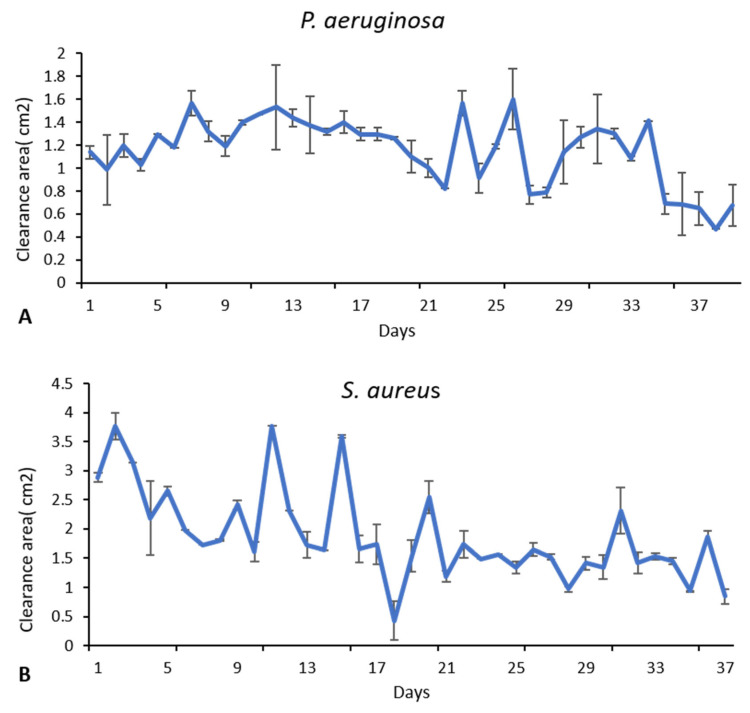
SRV-CHX and SRV-placebo coated sponges were daily transferred to new *S. aureus* (**B**) and *P. aeruginosa* (**A**) seeded agar plates, and the inhibition zone was monitored. The kinetic experiments revealed that SRV-CHX-coated sponges (Merocel) delayed bacterial growth of *S. aureus* for 30 ± 7 days and *P. aeruginosa* for 30 ± 9 days. SRV-placebo had no effect. The figures represent the average of 3 independent experiments. *p* < 0.05 for SRV-CHX-coated sponges when compared to SRV-placebo-coated sponges.

**Figure 4:**
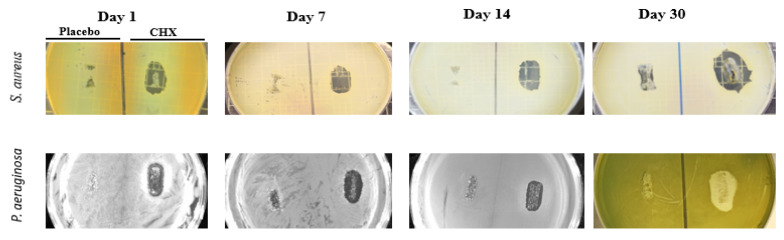
Representative images that show the inhibition zone around the sponge coated with SRV-CHX that has been plated on *S. aureus* and *P. aeruginosa* seeded agar plates. The control sponges coated with SRV-placebo did not cause any clearance of bacteria.

The SRV-CHX and SRV-placebo-coated sponges (Merocel) were incubated daily with freshly seeded bacteria on TSA plates for around 30 days. The area of the inhibition zone surrounding the samples was measured after an overnight incubation. Kinetic experiments demonstrated that the SRV-CHX-coated sponges (Merocel) effectively inhibited the growth of *P. aeruginosa* for 39 days (±SD) (Figure 3A and Figure 4), and *S. aureus* for 37 days (Figure 3B and Figure 4) and with a gradual decline in the size of the inhibition zone over time. Minor fluctuations observed in the inhibition-zone areas during the long-term kinetic assay are expected and can be attributed to small day-to-day variations in agar moisture, plate thickness, diffusion dynamics, and manual measurement variability. However, the overall trend demonstrates a clear and sustained inhibitory effect of SRV-CHX throughout the experimental period. In contrast, the SRV-placebo-coated sponge exhibited no inhibitory effect on bacterial growth. The effective period of the SRV-CHX-coated sponges extends far above the 3–5 days of the clinical use of the sponges.

We stopped the kinetic experiments after 39 days, even though the SRV-CHX-coated sponges still had some anti-bacterial activity, as our work is primarily aligned with clinical practice. In a clinical setting, the maximum duration for using sponges is typically 3 to 5 days, which makes it unnecessary to test for extended activity beyond this period.

### 3.2. Measuring the Antibacterial Effect of Coated Sponges (Merocel) on Planktonic Growth

**Figure 5:**
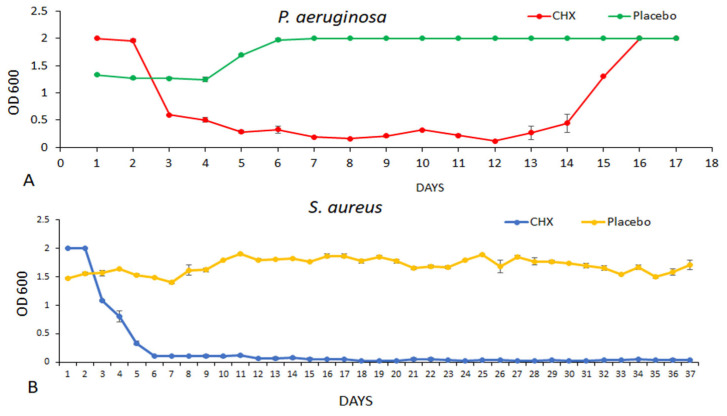
Antibacterial effect of coated sponges on planktonic growth. SRV-CHX and placebo-coated sponges were incubated in TSBG with bacteria, and OD was measured after 24 h. SRV-CHX-coated sponges significantly reduced bacterial growth of *P. aeruginosa* (**A**) and *S. aureus* (**B**) compared to SRV-placebo-coated sponges, which showed no effect on bacterial growth.

The SRV-CHX and SRV-placebo-coated sponges were daily incubated with fresh bacterial cultures of either *S. aureus* or *P. aeruginosa*, and the effect on planktonic bacterial growth was followed by measuring the OD. The OD of TSBG with SRV-CHX coated sponge (Merocel) in the absence of bacteria was used as a baseline for calculating the relative amount of bacteria in the culture after a 24 h incubation. Figure 5 indicates that the OD of the bacterial cultures that were incubated with sponges coated with SRV-CHX was significantly decreased for both *S. aureus* and *P. aeruginosa*, compared to those exposed to sponges coated with SRV-placebo, where the bacterial growth was unaffected. The high OD in the presence of the two bacterial species during the first two days is due to high concentrations of CHX released that precipitate proteins of the TSB medium and not of bacterial growth. This notion is supported by the drop method assay showing no bacterial growth on agar plates (Section 3.3).

There was a clear difference in the bacterial response to SRV-CHX among the two bacterial species. The antimicrobial effect on *P. aeruginosa* (about 14 days) was notably less effective compared to *S. aureus* extended over 37 days, indicating different sensitivity to the SRV-CHX of the two types of bacteria.

### 3.3. Determination of Bacterial Viability by Drop Plate Method

**Figure 6:**
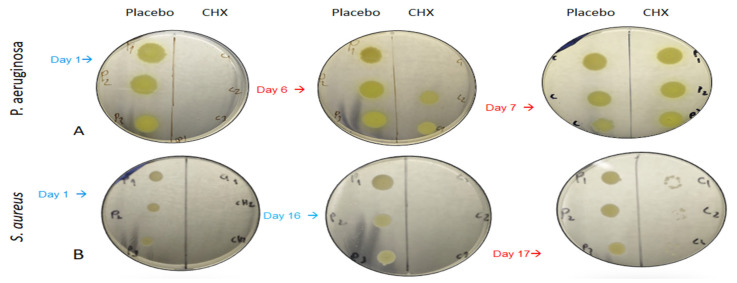
The bactericidal effect of the SRV-CHX-coated sponges was observed on *P. aeruginosa* (**A**) for 5 days and *S. aureus* (**B**) for 16 days. No effect was observed with the SRV-placebo on *P. aeruginosa* or *S. aureus*.

To further evaluate the antibacterial efficacy of SRV-CHX, two 10 µL drops of the bacterial cultures from both the SRV-CHX and SRV-placebo-exposed samples (Section 3.2) were placed on TSA plates and incubated for 24 h. The results indicated that SRV-CHX exhibited a bactericidal effect against *S. aureus* and *P.s aeruginosa*, lasting for 16 days and 5 days, respectively (Figure 6). Following this period, the antibacterial activity transitioned to a bacteriostatic mode, as evidenced by the appearance of bacteria on the agar plates (Figure 6) despite low turbidity (Figure 5). No anti-bacterial effect was observed with the SRV-placebo-coated sponges.

### 3.4. ATP Microbial Cell Viability Assay

**Figure 7:**
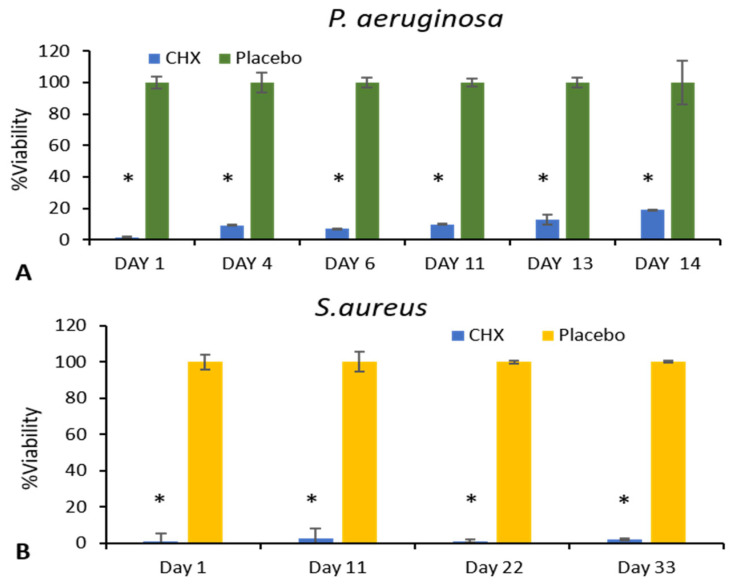
The BacTiter Glo microbial viability assay from selected days shows a significantly lower number of viable bacteria in the medium exposed to SRV-CHX-coated sponges for *P. aeruginosa* (**A**) and *S. aureus* (**B**), compared to the SRV-placebo. * *p* < 0.05.

To determine the relative number of viable bacterial cells, intracellular ATP levels were measured as a reliable marker of cell viability. ATP, the molecule responsible for cellular energy storage, is exclusively produced by living organisms, making it an effective indicator of bacterial activity and survival. In this experiment, SRV-CHX-coated sponges were incubated daily with fresh cultures of *P. aeruginosa* and *S. aureus*. The results showed a significantly lower number of viable bacteria in the cultures exposed to SRV-CHX-coated sponges compared to SRV-placebo-coated sponges. This reduction in bacterial viability persisted for 14 days for *P. aeruginosa* and up to 33 days for *S. aureus*, indicating that the sustained release of chlorhexidine from the coated sponges effectively inhibited bacterial growth over time (Figure 7). The continuous release of the antiseptic likely disrupted essential bacterial processes, reducing their ability to proliferate.

### 3.5. SRV-CHX-Coated Sponges Prevented Biofilm Formation for a Prolonged Time

#### 3.5.1. Inhibition of Biofilm Metabolic Activity

**Figure 8:**
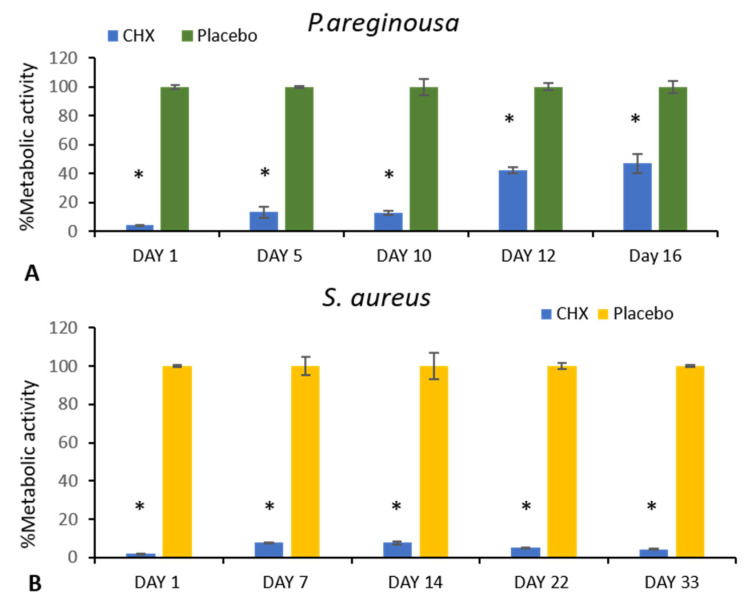
A significant reduction in metabolic activity of bacteria immobilized in biofilm formation, exposed to SRV-CHX-coated sponges, with suppression lasting 16 days against *P. aeruginosa* (**A**) and at least 33 days against *S. aureus* (**B**) as determined by using the MTT metabolic assay. * *p*-value < 0.05.

The metabolic MTT assay demonstrated a significant reduction of bacteria immobilized in biofilm formation surrounding the SRV-CHX-coated sponges compared to those coated with SRV-Placebo (Figure 8). This suggests that the SRV-CHX coating of sponges effectively inhibited biofilm formation for up to 10 days for *P. aeruginosa* (Figure 8A). However, after this period, the bacterial viability in the biofilms gradually increased to approximately 50% by day 16 compared to the SRV-placebo. In contrast, SRV-CHX-coated sponges inhibited *S. aureus* metabolic activity of bacteria of biofilm formation for the entire test period of 33 days (Figure 8B).

#### 3.5.2. Biofilm Biomass

**Figure 9:**
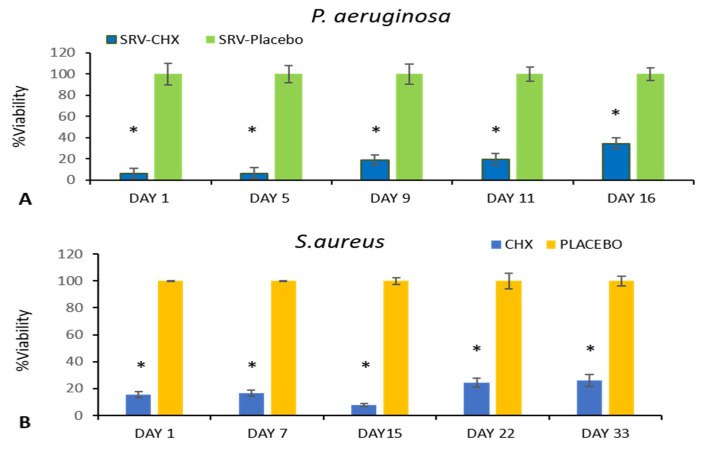
A significant reduction in biofilm formation was observed in the medium treated with SRV-CHX, with suppression lasting for 16 days against *P. aeruginosa* (**A**) and extending up to 33 days against *S. aureus* (**B**). The experiments for OD, MTT, CV, and ATP tests were stopped after 33 days, as the extended duration surpassed the clinical requirements of 3 to 5 days, which is the typical timeframe for such treatments, as previously explained. * *p*-value < 0.05 compared to SRV-placebo-coated sponges.

The Crystal Violet (CV) staining assay further validated the efficacy of SRV-CHX in preventing biofilm formation. Results showed a significant reduction in biofilm development in the medium surrounding the SRV-CHX-coated sponges, compared to the SRV-placebo-coated sponges. Specifically, the SRV-CHX treatment prevented biofilm formation for up to 10 days in *P. aeruginosa*; thereafter, the bacterial viability increased to approximately 50% of that of SRV-placebo treatment by day 16 (Figure 9A). In contrast, *S. aureus* displayed no recovery of bacterial viability, with biofilm formation effectively inhibited for 33 days, as illustrated in Figure 9B.

#### 3.5.3. High-Resolution-Scanning Electron Microscope (HR-SEM) Evaluation of Biofilm Formation

**Figure 10:**
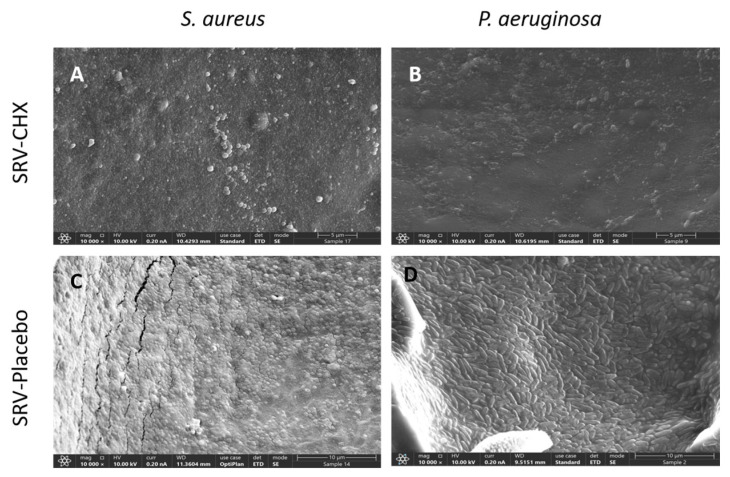
HR-SEM images of SRV-CHX-coated sponges (**A**,**B**) and SRV-placebo-coated sponges (**C**,**D**) after 14 exposures to planktonic *S. aureus* (**A**,**C**) or *P. aeruginosa* (**B**,**D**) are shown at ×10,000 magnification.

The HR-SEM images revealed a significant reduction in biofilm formation on sponges coated with SRV-CHX for both bacterial strains, compared to sponges coated with SRV-placebo (Figure 10). Continuous biofilms of *S. aureus* and *P. aeruginosa* were evident on the SRV-placebo-coated sponges (Figure 10C,D), whereas only scattered clusters of *S. aureus* and isolated *P. aeruginosa* cells were detected on the SRV-CHX-coated sponges (Figure 10A,B). These findings demonstrate visually that SRV-CHX coating of sponges significantly inhibits biofilm formation.

#### 3.5.4. Live/Dead Staining on Spinning Disk Confocal Microscopy (SDCM)

**Figure 11:**
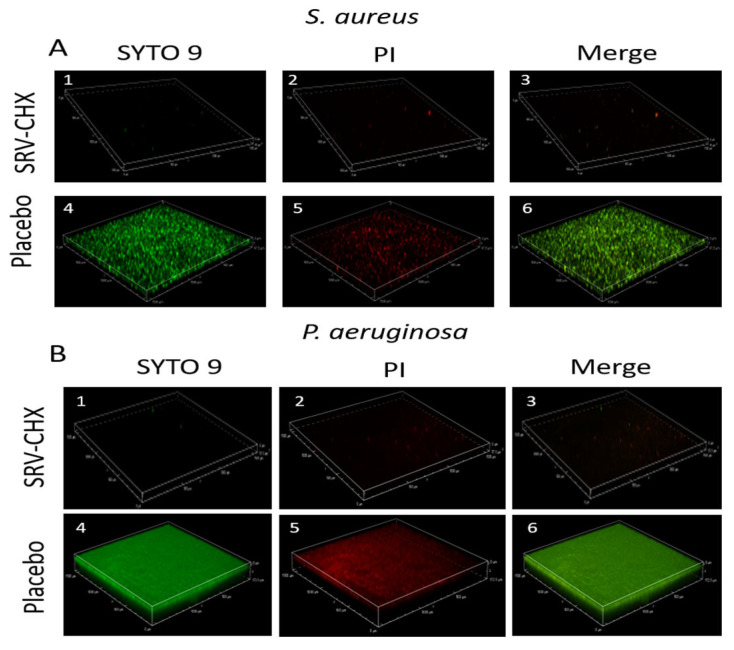

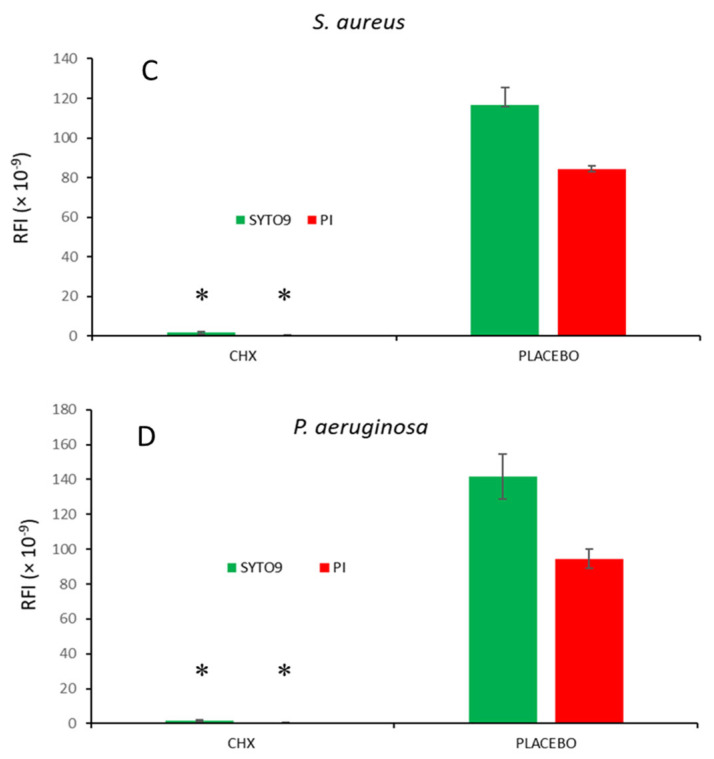
(**A**,**B**) SYTO 9/PI staining of *S. aureus* (**A**) and *P. aeruginosa* biofilms (**B**) formed during the 7th exposure to bacterial culture in the presence of SRV-CHX-coated (subfigures (A1–3, B1–3)) or SRV-placebo (subfigures (A4–6, B4–6)) Merocel sponges. The biofilms were visualized by a Nikon eclipse Ti-U confocal spinning disk microscope. (**C**,**D**) Quantification of the relative fluorescence intensities (RFI) of SYTO 9 (green columns) and PI (red columns) in biofilms shown in A and B. * *p* < 0.05. The images cover a biofilm area of 1500 µm × 1500 µm.

After a 7-day incubation of SRV-coated Merocel sponges exposed to bacterial cultures of *S. aureus* or *P. aeruginosa*, the biofilm formation on the surface around the Merocel was analyzed using dead/live SYTO 9/PI staining, visualized by spinning disk confocal microscopy (SDCM). SDCM images of biofilms formed by *S. aureus* and *P. aeruginosa* after 7 days of exposure to SRV-placebo-coated sponges displayed strong SYTO 9 (green) fluorescence with sparse PI (red) fluorescence (Figure 11(A4,5,B4,5)), indicating a dense biofilm layer composed primarily of live bacteria. In contrast, the SRV-CHX-coated Merocel exhibited scattered green fluorescence alongside predominant red fluorescence, suggesting that most of the bacteria attached to the surface were dead (Figure 11(A1,2,B1,2)). This visual impression is corroborated by the quantitative analysis of fluorescence intensities (Figure 11C,D), which shows a significantly higher proportion of PI signal in the SRV-CHX group compared with the SRV-placebo group (*p* < 0.05).

## 4. Discussion

The non-absorbable sponge (Merocel) has been widely used in otolaryngology for managing acute and chronic otitis externa, necrotizing otitis externa, and nasal bleeding (1–3). Despite its clinical utility, Merocel sponges are susceptible to bacterial colonization and biofilm formation, which can lead to complications such as chronic rhinosinusitis and, in rare cases, toxic shock syndrome [8,14]. The present study demonstrated that coating Merocel with a sustained-release varnish containing chlorhexidine (SRV-CHX) significantly inhibited bacterial growth and biofilm formation. This approach represents a novel antimicrobial strategy that may reduce reliance on systemic antibiotics while supporting antimicrobial stewardship and improving patient safety.

Chlorhexidine (CHX) is a well-established antiseptic with documented safety in nasopharyngeal gels [25], dermatologic formulations [26], and various medical devices [17,18,19]. Incorporating CHX into a sustained-release varnish enables continuous local antibacterial activity, limiting microbial colonization while reducing the likelihood of resistance development. These characteristics make SRV-CHX an attractive candidate for clinical applications that require prolonged antimicrobial protection.

In clinical practice, Merocel ear wicks and nasal sponges are typically retained for 3–5 days [27,28], during which local and systemic antibiotics are often prescribed to prevent infections and complications such as sinusitis and toxic shock syndrome [13]. The current findings suggest that SRV-CHX-coated sponges may provide extended local antimicrobial protection, thereby reducing the need for systemic antibiotics. This innovation could enhance outcomes in otitis externa and in patients requiring nasal packing after surgery, while minimizing antibiotic-related side effects and resistance.

The antibacterial efficacy of SRV-CHX was robust. Disk diffusion assays demonstrated prolonged inhibition of *S. aureus* (37 days) and *P. aeruginosa* (39 days)—two clinically relevant pathogens associated with otitis externa and nasal infections. Placebo-coated sponges exhibited no inhibitory effect, underscoring the essential role of SRV-CHX in achieving sustained antimicrobial activity. The results above indicate that the SRV-CHX has a gradual, prolonged release of antibacterial/antibiofilm properties. The different assays describe different properties of the SRV-CHX as an antibacterial agent. Figure 6 shows an effect on agar plates, where the diffusion through the agar is a rate-limiting factor in the release kinetics. Figure 5 demonstrates the release rate in a planktonic environment. Figure 7 indicates metabolomic effects as measured by ATP, while Figure 8 demonstrates metabolomic inhibition in biofilm conditions, and Figure 9 describes the inhibitory effect of biofilm biomass.

Planktonic bacterial assays confirmed that SRV-CHX markedly reduced bacterial growth for both *S. aureus* and *P. aeruginosa*, indicating effective suppression of bacterial proliferation. Biofilm metabolic assays further revealed significant reductions in bacterial activity within biofilms formed on SRV-CHX-treated sponges. Because biofilm-associated infections are notoriously resistant to conventional antibiotics, these results emphasize the clinical importance of this approach. The shorter effective inhibition period observed for *P. aeruginosa* compared with *S. aureus* is consistent with the higher intrinsic tolerance of *P. aeruginosa* to cationic antiseptics such as CHX, which is attributed to its outer-membrane barrier, efflux systems, and biofilm-associated protective factors.

Microscopic analyses provided mechanistic insights. High-resolution scanning electron microscopy (HR-SEM) showed minimal bacterial accumulation and an absence of extracellular polymeric matrix on SRV-CHX-coated sponges, in contrast to the dense biofilms observed on placebo controls. Spinning disk confocal microscopy (SDCM) confirmed that most bacterial cells on SRV-CHX-treated sponges were non-viable, visually validating the varnish’s potent bactericidal effect.

The kinetic diffusion experiments were concluded for several weeks, depending on the bacteria and the analytical methodology. The duration of the release is associated with the clinical relevance. In practice, sponges are typically used for a maximum of 3–5 days. The extended period of release we have found is important in the biological characterization of this new type of coating. In the above long-term in vitro experiments, the gradual reduction in antibacterial activity over time reflects depletion of freely diffusible, biologically active CHX from the varnish. CHX is a di-cationic stable molecule that, at high concentrations, may interact with proteins and organic components in media and thereafter lose some of its activity. Our biological assay clearly shows that its antibacterial/antibiofilm activity is maintained for several weeks.

Although we did not directly measure CHX release, the microbial release profiles are as follows: prolonged inhibition zones, reduced planktonic growth, and sustained suppression of biofilm activity clearly indicate continuous release from the SRV-CHX coating. The proof of concept that the coated sponge can affect bacteria is of clinical importance. In order to detect the pharmaceutical release profiles, a study in sink conditions detecting the amount of released CHX should be conducted in the future.

The observed prolonged efficacy of SRV-CHX corresponds with the findings of the MTT metabolic assay (Section 3.5.1), which demonstrated consistent suppression of bacterial viability. These results align with previous reports describing the long-term antibacterial performance of SRV-CHX in other otolaryngologic devices, including sino-nasal stents [17] and voice prostheses [18]. Collectively, the evidence supports the potential of SRV-CHX-coated Merocel sponges to prevent biofilm-associated infections, enhance clinical outcomes, and reduce dependence on systemic antibiotics in both ear and nasal applications.

This study was conducted entirely in vitro, which limits direct extrapolation to clinical practice. Although antimicrobial efficacy was sustained for several weeks, the experiments did not include direct assessment of chlorhexidine release kinetics or cytotoxicity toward host tissues. Antimicrobial activity was therefore used as a surrogate indicator for drug release. Moreover, only two bacterial species were tested, whereas clinical infections involving otitis externa and nasal packing are often polymicrobial. Future in vivo studies and pharmacokinetic evaluations are warranted to confirm the safety, tolerability, and release characteristics of SRV-CHX-coated sponges in clinical settings.

## 5. Conclusions

This study demonstrated that coating non-absorbable sponges (Merocel) with a sustained-release varnish containing chlorhexidine (SRV-CHX) effectively inhibited bacterial growth and biofilm formation. SRV-CHX-coated sponges exhibited prolonged antimicrobial activity, showing sustained inhibition of both *Staphylococcus aureus* and *Pseudomonas aeruginosa* for several weeks, with complete prevention of biofilm accumulation for more than 30 days. These findings highlight the durable antibacterial and anti-biofilm efficacy of SRV-CHX and suggest a promising strategy to reduce dependence on systemic and topical antibiotics, improve infection control, and enhance patient outcomes.

Future studies are warranted to validate these findings in vivo, establish detailed drug-release profiles, and confirm clinical safety and performance. The present in vitro results provide a strong foundation for developing next-generation antimicrobial packing materials in otolaryngology.

## Data Availability

The raw data supporting the conclusions of this article will be made available by the authors on request.
